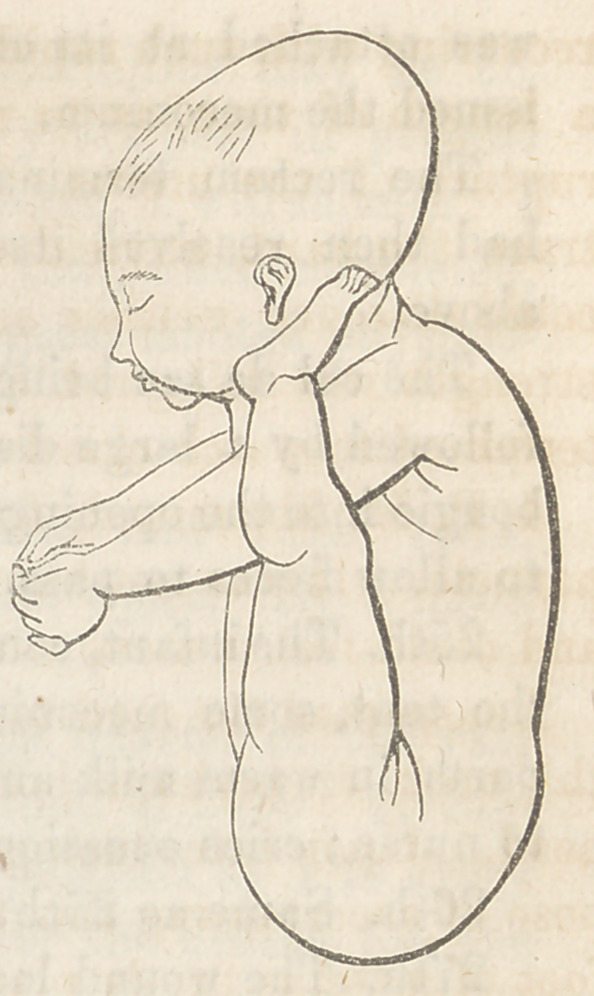# Singular Fœtal Malformation

**Published:** 1854-08

**Authors:** N. C. Skinner

**Affiliations:** North Carolina


### *Singular Fœtal Malformation.*

(Communicated to John Wiltbank, M. D.)

By N. C. SKINNER, M. D., of North Carolina.

Mrs. Ch. B. of Chester County, Penna., aged 27 years, of spare habit, good general health, was taken in labor with her second child, at 7, A. M., June 23d, 1853. I saw her at 8½, A. M. Found her depressed in spirits, apprehensive "that all was not right;" has felt slight labor pains for the last twenty-four hours.

She stated to me that she had been very anxious concerning herself for several months past, and that towards the latter part of her pregnancy this anxiety had increased to such a degree as to render her desirous of consulting me, but that she was prevented by her friends, who ridiculed the necessity.

Upon inquiry as to the cause of her anxiety, she said that some four months ago, whilst her husband was engaged in a fight, she became suddenly alarmed at seeing him fall, and about to be struck with an axe. She then felt for the first time, several vigorous movements of the fœtus, accompanied with a slight discharge of blood from the uterus, and a faintness. Ever after this, the movements of the fœtus were feeble, with long intervals of time elapsing between them, "long enough to make her suppose her child to be dead."

The abdomen presented the usual appearance of a woman at full term. The pains were natural, both in force and frequency. Vaginal examination discovered the os tincæ dilated to the size of a Spanish quarter, thick and rather unyielding; felt a hard round tumor high up in the superior strait, and became satisfied of a cranial presentation.

11 A. M. Pains recurring at short and regular intervals, and with more than ordinary severity; examined again and found the head advanced but very little; os more dilated and yielding; membranes protruding.

1 P. M. The pains had now become very rapid and intensely severe. I ruptured the bag of waters; not over half a pint escaped. At the next and for several successive pains, the head slowly descended until it was about to engage the inferior strait, where it became arrested.

2½ P. M. During the last hour, the labor pains had been recurring with such rapidity and violence, and continued so great a length of time, as to make me seriously apprehensive of a rupture of the uterus. Upon examination, I could distinctly trace the hard, unyielding globe, like the head of a fœtus, but could discover neither suture nor fontanelle. As the patient's strength was still good, and other circumstances favorable, I determined to watch closely her unassisted efforts.

4 P. M. Examined, found the head had not advanced one particle. It was not impacted, could raise it up, (ballottement,) and could feel it move with the motions of the fœtus.

I suspected the cord to be abnormally short, or twisted around the neck, or perhaps both, thus preventing the descent of the head. Upon introducing my hand into the vagina, for the purpose of ascertaining this fact, my fingers came in contact with a foot of a fœtus firmly planted below the ear, behind the angle of the lower jaw. I did not detect the cord twisted around the neck. The idea of twin pregnancy occurred to me, occasioned by the discovery of a foot in such an awkward position. The examination also showed an occipito-sacral presentation of a head completely ossified and solidified, and of rather more than ordinary size.

9 P. M. The patient's strength now rapidly failing, without any prospect of a speedy delivery, and as she had also become greatly alarmed and excited, I emptied the rectum and bladder; administered chloroform by inhalation to complete anæsthesia, and delivered slowly and carefully with forceps at 10 P. M. In 15 minutes delivered placenta, which was slightly adherent.

The funis, which I send you, was most strangely knotted into a single tie, and an additional double sailor's knot.

The patient was fifteen hours in active labor, during ten of which the pains were alarmingly severe. The head of this fœtus presented nothing unusual in form, except rotundity. It was rather above the ordinary size, and completely solidified; both fontanelles and each suture being firmly and smoothly ossified. There was osseous anchylosis of the ankle, knee and hip joints; in fact, the bones of the legs from the toes to the hip, seemed each to be formed of a continuous straight bone, with but little enlargment to indicate where nature intended to form joints.

The soles of each foot were firmly planted against the neck and lower jaw, immediately beneath each ear, (but not adherent thereto,) and it required considerable force to remove them one inch either way.

The arms stood out at right angles with the body, but they could, by a little exertion, be brought down to their natural place; the moment, however, they were freed from restraint they would fly back with a spring to their unnatural position. The features were natural in appearance, the trunk plump, round and slightly curved forwards.

Not the least extraordinary deformity of this fœtus, consisted in the eccentric termination of the rectum. Being imperforate, I proceded in eighteen hours after birth to operate for artificial anus.

I could perceive no distension of membrane, when the child was made to cry, in order the better to observe the spot where the rectum terminated. Using a bistoury at the spot where the rectum should naturally have terminated, I made an incision ¼ inch deep, but no meconium followed. Through the opening I had made, I then introduced my finger, well oiled, and with the handle of a scalpel dissected up nearly two inches in search of the cul de sac of the rectum, with the view of opening it, for such I had conceived to be its termination. My finger here came in contact with an elastic bladder-like tumor, which proved to be the abrupt termination of the rectum, drawn forward somewhat out of its natural position.

Upon pressing upon the sac, I was surprised to see a small drop of meconium, larger than the head of a small pin, issue from a point anterior to and in a line with the raphe of the scrotum. Clearing this drop away, and again pressing upon the the cul de sac, another small drop issued at the same point, from a minute opening, not capable of allowing the introduction of a fine needle. Upon a more careful examination of the cul de sac, I discovered attached to its anterior side a ligamentous cord, which was attached at its other extremity to the spot from whence issued the meconium.

The rectum terminated at the sigmoid flexure high up, and had then resolved itself into this small cord, which ended as above.

The cul de sac being now opened with a straight bistoury, was followed by a large discharge of meconium. Introduced a wax bougie into the opening, with directions to remove it in eight hours to allow fæces to pass.

25th. The infant, contrary to expectation, was living; removing the tent, some meconium and fecal matter passed; washed the parts in warm milk and water; has not yet shown a disposition to nurse; cries occasionally. The mother doing well.

26th. Same as 25th. Passed urine per vias naturales.

27th. The wound looks unhealthy, fæces still pass out through it; no disposition to nurse; some febrile disturbance.

28th. Same.

29th. Considerable general disturbance. Died at 11 P. M., 7 days after birth.

Owing to the very great (and natural) prejudices of the parents, I was unable to procure the specimen for preservation; an examination was not even permitted. The accompanying drawing will, however, give an accurate representation of the fœtus, the rectum and funis.

In regard to the cause of this malformation, is it not reasonable to suppose, after having carefully weighed the data furnished by the mother, that the foundation of the mischief was laid at the moment she experienced the shock at seeing her husband placed in a dangerous situation? And that her imagination, and the constant anxiety with which she was harassed, that "all was not right," kept and maintained the fœtus in its awkward position to the end of fœtal life? I do not mean to convey the idea that mechanical force was exerted to produce the effect. But that the same principle which causes "the babe to leap in its mother's womb," in response to any emotion on her part, not cerebro-spinal, would continue to exert its influence for a time sufficiently long and powerful, as to be productive of such physical deformity.

A table of monstrosities, exhibiting their peculiarities and varieties, together with a brief history of events occurring to the mother during gestation, by which the deformities may be accounted for, is now and has been for two years past in process of compilation by myself, which, when finished, I will submit. I am encouraged in this work by the hope, that, when completed, it may have some tendency to establish a great mooted physiological point.

## Figures and Tables

**Figure f1:**